# Reducing sitting time is essential for preserving sarcopenia and functional independence with aging: a comparative cross-sectional study

**DOI:** 10.7717/peerj.21714

**Published:** 2026-09-08

**Authors:** Tae-Hyun Yoon, Yong-Seok Jee

**Affiliations:** Research Institute of Sports and Industry Science, Hanseo University, Seosan, Chungcheongnamdo, Republic of South Korea

**Keywords:** Sitting time, Sarcopenia, Functional independence, Physical performance, Older adults

## Abstract

**Background:**

As individuals age, sitting time is increasingly recognized as a factor that undermines independence in daily living; however, the extent to which sitting time influences sarcopenia-related indicators and functional independence remains unclear.

**Methods:**

A total of 200 community-dwelling older adults aged 65–96 years were categorized into three groups based on daily sitting duration: low (<6 h/day), moderate (6–8 h/day), and high (>8 h/day) groups. Sarcopenic measures—including calf circumference, grip strength, 5-time chair stand test, and appendicular skeletal muscle mass (ASM)—were evaluated. Functional independence was examined using assessments of activities of daily living (ADL) and instrumental activities of daily living (IADL). Analysis of covariance (ANCOVA) was used to examine group differences across sitting time categories with adjustment for age and sex. Spearman’s correlation analysis was performed to examine associations between sitting time and sarcopenia-related indicators and functional independence variables, and separate simple linear regression were subsequently constructed with sitting time entered as the independent variable and sarcopenia-related indicators and functional independence variables included as dependent variables.

**Results:**

Calf girth differed significantly by sex (η^2^ = 0.995) and group (η^2^ = 0.362). Grip strength showed a significant sex-by-group interaction (η^2^ = 0.068), with a similar pattern observed for 5-time chair standing (η^2^ = 0.069). ASM was primarily influenced by age (η^2^ = 0.160), with no significant group or sex effects. Increasing sitting time was associated with greater functional decline in mobility-related ADL items, remaining significant after adjustment for age (η^2^ = 0.077) and sex (η^2^ = 0.286). Higher sitting time was also linked to poorer higher-level IADL functions (telephone use, housekeeping, transportation, and financial management), independent of age (η^2^ = 0.023) and sex (η^2^ = 0.243). ADL and IADL were strongly correlated (ρ = 0.812) and were significantly associated with sarcopenic indicators, including calf girth, grip strength, chair stand performance, and ASM. In regression analysis, sitting time was negatively associated with ASM (β = −0.415, *p* < 0.001), calf circumference (β = −0.442, *p* < 0.001), and grip strength (β = −0.385, *p* < 0.001), and positively associated with 5-time chair standing test (β = 0.465, *p* < 0.001), indicating poorer lower-extremity physical performance. In addition, sitting time was negatively associated with ADL sum score (β = −0.308, *p* < 0.001) and IADL sum score (β = −0.410, *p* < 0.001).

**Conclusion:**

These findings suggest that longer sitting time may be broadly related to sarcopenia-related indicators and daily functional independence. However, because this study employed a cross-sectional design, these relationships should be interpreted as associations rather than causal effects.

## Introduction

Sitting duration, typically defined as waking activities performed in a seated or reclined posture with low energy expenditure, has become increasingly prevalent among older adults and is now recognized as a distinct behavioral risk factor for adverse health outcomes (Owen et al., 2011; Tremblay et al., 2017). Prolonged sitting time, as a key component of sedentary behavior, has been associated with cardiometabolic diseases, frailty, and increased mortality, independent of moderate-to-vigorous physical activity (PA) levels (Biswas et al., 2015; Ekelund et al., 2016). As aging is accompanied by reductions in physiological reserve, older adults may be particularly vulnerable to the negative consequences of sustained sitting time.

One important pathway through which sitting time may influence health in older adults is *via* sarcopenia, a progressive and generalized skeletal muscle disorder characterized by declines in muscle mass and muscle strength, with physical performance considered an important functional outcome related to sarcopenia severity and progression according to recent updates of the Asian Working Group for Sarcopenia (AWGS) framework (Chen et al., 2020; Cruz-Jentoft et al., 2019). Sarcopenia is strongly associated with falls, disability, loss of independence, and mortality (Beaudart et al., 2017; Landi et al., 2018). Experimental and observational studies suggest that prolonged muscle unloading and reduced neuromuscular activation associated with sitting time may accelerate muscle atrophy, impair muscle quality, and compromise physical performance, thereby contributing to sarcopenic impairments (Breen & Phillips, 2011).

Functional independence is a critical determinant of healthy aging and is commonly assessed using activities of daily living and instrumental activities of daily living. Activities of daily living (ADL) reflect basic self-care abilities, whereas instrumental activities of daily living (IADL) capture more complex functions required for independent community living, such as shopping, transportation, and financial management, and declines in these functional domains are associated with poorer quality of life, increased healthcare utilization, and a higher risk of institutionalization (Lawton & Brody, 1969; Gill et al., 2010).

Although accumulating evidence links sitting time to reduced physical function, most previous studies have focused on global physical performance or mobility outcomes, with limited attention to specific sarcopenic parameters and different levels of functional independence. Moreover, although previous studies, including a recent systematic review and meta-analysis (Mo et al., 2023), have reported associations between sedentary behavior and sarcopenia in older adults, it remains less clear how daily sitting time is related to multiple sarcopenia-related indicators together with different levels of functional independence, particularly ADL and IADL, in community-dwelling populations. Clarifying these associations is essential for identifying behavioral targets that may help preserve muscle health and functional independence in aging populations. In other words, although prolonged sitting has emerged as a major risk factor contributing to functional decline in older adults, the extent to which sitting time affects sarcopenic indices and functional independence, as well as the strength and nature of the interrelationships among these variables, remains inconclusive. Therefore, this study aimed to investigate whether daily sitting time is associated with sarcopenia-related indicators and functional independence in community-dwelling older adults. In particular, we examined cross-sectional associations between sitting time and appendicular skeletal muscle mass, muscle strength, physical performance, and independence in both basic and instrumental activities of daily living.

## Materials and Methods

### Study design and participants

This study employed an observational cross-sectional design. Community-dwelling older adults aged ≥65 years were recruited from a local community facility (Seoul Seniors Tower) in Seoul, Korea, between November 1, 2024, and February 28, 2025. Eligible participants were individuals who could walk independently with or without assistive devices, had not participated in supervised exercise or rehabilitation programs for more than 6 months, and were able to complete all required physical tests and self-report questionnaires. In addition, eligibility criteria were restricted to non-smokers and individuals who did not consume alcohol at the time of participation. Participants were excluded if they had acute musculoskeletal injuries, severe neurological disorders affecting mobility, unstable cardiovascular conditions, or cognitive impairment that precluded reliable participation in the study procedures.

Sample size estimation was conducted using G*Power version 3.1 (Kang, 2021) based on a multiple regression framework in which sarcopenic parameters were specified as predictors of functional independence across levels of sitting time to ensure adequate statistical power. Assuming a small-to-moderate effect size (f^2^ = 0.10), an α level of 0.05, and a statistical power of 0.80, the minimum required sample size was estimated to be 114 participants. To account for potential missing data and to ensure sufficient power for nonparametric group comparisons and item-level analyses, a larger sample size was targeted (Sedgwick, 2013). The final sample size exceeded this minimum requirement, providing adequate power for the primary analyses.

### Measurement methods

#### Demographic and daily life characteristics

Demographic characteristics included age, sex, monthly living expenses, residential area, educational attainment, marital status, living arrangement, self-rated health status, and chronic disease (Yaghjyan et al., 2019). Monthly living expenses were converted from Korean won (KRW) to U.S. dollars (USD) using an approximate average exchange rate of 1 USD = 1,420 KRW during the study period to enhance international comparability. The converted monthly expenditure categories were defined as follows: <$760, $770–1,140, $1,150–1,530, $1,540–1,920, $1,920–2,300, and ≥$2,300. Residential areas were classified as either rural or urban. Educational attainment was categorized as: illiterate, no formal schooling but literate, elementary school graduate, middle school graduate, high school graduate, university graduate, and graduate school or higher. Marital status was classified as having a living spouse or being widowed. Living arrangement was categorized as living alone or living with family. Self-rated health status was assessed on a five-point scale: very good, good, fair, poor, and very poor. Finally, physician-confirmed chronic diseases were assessed using a multiple-response approach.

Among daily life variables, in-bed time was determined by recording the time of sleep onset and calculating the interval until awakening the following morning and resumption of usual daily activities, which was operationally defined as in-bed time. Daily caloric intake was estimated using a standardized dietary framework developed by a certified dietitian that included both macronutrient and micronutrient components. Participants recorded their habitual food consumption under free-living conditions, including meals consumed outside the standardized meal framework. All dietary records were entered and analyzed by a single trained researcher using CAN-Pro 5.0 software (Korean Nutrition Society; https://www.kns.or.kr/can/CanPro5.asp) to ensure consistency in data processing. The standardized diet therefore served as a reference framework for estimating daily intake rather than as a controlled dietary intervention protocol. In the present study, daily sitting time was assessed using the sitting-time item of the International Physical Activity Questionnaire–Short Form (IPAQ-SF), which is a widely used and validated instrument for estimating sedentary behavior in adult populations. Total physical activity was calculated in metabolic equivalent hours per week (MET·h/week) according to the standardized scoring protocol of the IPAQ-SF (Chun, 2012).

#### Sitting time

Sitting time was primarily assessed using self-reported daily sitting time, which reflects the total amount of time spent sitting or reclining during waking hours. Average daily sitting time was assessed using the sitting-time item of the IPAQ-SF. Participants reported the total time spent sitting on a typical weekday during the previous week, and trained researchers administered the questionnaire in an interviewer-assisted format to ensure completeness and accuracy of responses. To complement self-reported data and provide an objective proxy of daily movement behavior, participants’ smartphone-based walking and activity tracking applications were additionally used to record daily step counts and active time. Trained researchers assessed and analyzed participants’ average daily sitting time (hours/day) over a typical week. Based on reported information, the cut-off values have been commonly used in epidemiological studies to distinguish sedentary risk levels in older adults and to facilitate interpretation of dose–response relationships between sitting time and health outcomes. Based on previous literatures (Biswas et al., 2015; Chau et al., 2013; Ekelund et al., 2016), <6 h/day was classified as low sitting time, 6–8 h/day as moderate sitting time, and >8 h/day as high sitting time.

#### Sarcopenia and body composition measures

Sarcopenia was assessed in accordance with the criteria recommended by the AWGS guidelines (Chen et al., 2020). Calf circumference was measured bilaterally using a non-elastic tape positioned at the point of maximal calf girth without compressing the subcutaneous tissue according to a standardized protocol (Bonnefoy et al., 2002), the mean value was used for analysis, and low calf circumference was defined as <34 cm for men and <33 cm for women according to the AWGS 2019 criteria. Grip strength was assessed using a handgrip dynamometer (TKK 5401; Takei Scientific Instruments Co., Ltd., Tokyo, Japan), with participants instructed to hold the device with the arm positioned approximately 30° away from the body and to exert maximal force; two trials were performed for each hand, the mean value was used for analysis, and low grip strength was defined as <28 kg for men and <18 kg for women. Physical performance was assessed using the 5-time chair stand test, which measures the time required to rise from a seated position five consecutive times with the arms crossed over the chest, and a completion time of ≥12 s was applied as the cutoff value for both men and women. Appendicular skeletal muscle mass (ASM) was measured using dual-energy X-ray absorptiometry (DXA; Lunar Co., Madison, WI, USA), and skeletal muscle index (SMI) was calculated by summing lean mass from the upper and lower extremities and normalizing it to height squared (kg/m^2^). Low ASM was defined as <7.0 kg/m^2^ for men and <5.4 kg/m^2^ for women according to the AWGS 2019 criteria (Chen et al., 2020). Additional body composition parameters, including body weight, lean mass, fat mass, and body mass index (BMI), were assessed according to standardized DXA-based procedures (Heo & Jee, 2024), and standing height was measured using a calibrated anthropometer (BMS 330; BioSpace Co., Ltd., Seoul, Korea).

#### Assessment of ADL of functional ability

The Barthel Index was selected to assess ADL because it provides a continuous measure of functional ability with greater sensitivity to subtle functional changes compared with dichotomous ADL instruments such as the Katz Index (Hartigan, 2007), making it more suitable for mediation analysis in older adults. This instrument consists of 10 items assessing basic self-care and mobility functions, including eating, transferring from bed to chair, personal toileting, toilet use, bathing or showering, walking along a corridor, ascending and descending stairs, dressing, bowel continence, and urinary continence. These items reflect fundamental physical self-maintenance abilities and identify functional limitations in daily living (Roehrig et al., 2007). Each item was scored dichotomously as either independent or requiring assistance, with full credit awarded only when the participant was able to perform the activity independently. A total ADL score was calculated by summing the scores across all 10 items. Participants were subsequently classified as without functional limitations if they achieved the maximum total score and as with functional limitations if their total score was below 100%. The ADL questionnaire demonstrated excellent internal consistency in this study, as indicated by Cronbach’s α coefficient of 0.894.

#### Assessment of IADL of functional ability

IADL was assessed using the Lawton and Brody scale. Although each item was scored dichotomously, a summed score (range: 0–8) was used as a continuous variable to preserve variability and enhance statistical power in the correlation and regression analyses (Lawton & Brody, 1969; MacCallum et al., 2002). This questionnaire consists of eight items evaluating higher-level functional abilities required for independent living, including the ability to use the telephone, shopping, food preparation, housekeeping, laundry, traveling by car or public transportation, medication management, and handling finances. The IADL scale extends the assessment of basic ADL by capturing everyday functional competence and the capacity to adapt independently to the environment (Garman & Cohen, 2002; Spector et al., 1987). A total IADL score was calculated by summing the scores across all eight items, and participants were classified as having no functional limitations if they achieved the maximum score of eight points, whereas those with a total score <8 or requiring assistance in at least one item (score = 0) were categorized as having IADL limitations. The internal consistency of the IADL questionnaire was high, as indicated by Cronbach’s α coefficient of 0.938.

### Ethics

This study was conducted in accordance with the ethical principles outlined in the Declaration of Helsinki. The study protocol was reviewed and approved by the Institutional Review Board of Hanseo University (HS24-10-22). Prior to participation, all participants were provided with a detailed explanation of the study procedures, potential risks, and benefits, after which written informed consent was obtained from each participant, and they were assured that their data would remain confidential and anonymous and would be used solely for research purposes.

### Statistical analysis

All statistical analyses were performed using IBM SPSS Statistics version 27.0 (IBM Corp., Armonk, NY, USA) and GraphPad Prism 10.6.1 (La Jolla, CA, USA). In this study, nominal variables were summarized as counts (*n*), continuous variables as mean ± standard deviation (SD), and categorical variables as frequencies and percentages [*n* (%)]. Differences between groups for categorical variables were assessed using the χ^2^ test or Fisher’s exact test, as appropriate. Given the cross-sectional design, analytical methods accounted for potential confounding by adjusting for age and sex. The prevalence of physician-diagnosed chronic diseases was analyzed by sitting time categories using a 10 × 10 dot plot to display frequencies (Rodrigues & Weiskopf, 2018). The normality of continuous variables was assessed using the Kolmogorov–Smirnov test. Prior to the main analyses, differences in participant characteristics among the three groups were examined using the Kruskal–Wallis test. Analysis of covariance (ANCOVA) was performed to compare body composition and sarcopenic parameters across sitting time categories while adjusting for age as a covariate and including sex as a fixed factor due to its categorical nature. To control for potential alpha-error accumulation due to multiple comparisons, Bonferroni-adjusted *post hoc* tests were applied where appropriate. Spearman’s correlation (ρ) analysis was conducted to examine bivariate associations between sarcopenia components and measures of functional independence, including ADL and IADL. To examine the associations between sitting time and sarcopenia-related indicators and functional independence, separate simple linear regression analyses were performed. Sitting time was entered as the independent variable, while calf circumference, grip strength, 5-time chair stand performance, ADL sum score, and IADL sum score were entered as dependent variables in separate models. Regression coefficients (B), standard errors (SE), standardized regression coefficients (β), and coefficients of determination (R^2^) were reported to evaluate the strength of associations. Effect sizes were interpreted using partial eta squared (η^2^), with values of 0.01, 0.06, and 0.14 representing small, medium, and large effects, respectively. Meanwhile, to quantify the additional variance explained by individual predictors—that is, the key indicators in the regression analysis—Cohen’s f was applied, with values of 0.02, 0.15, and 0.35 representing small, medium, and large effects, respectively (Cohen, 1992). Statistical significance was set at *p* ≤ 0.05 for all analyses.

## Results

### Demographic and anthropometric characteristics

**Figure 1:**
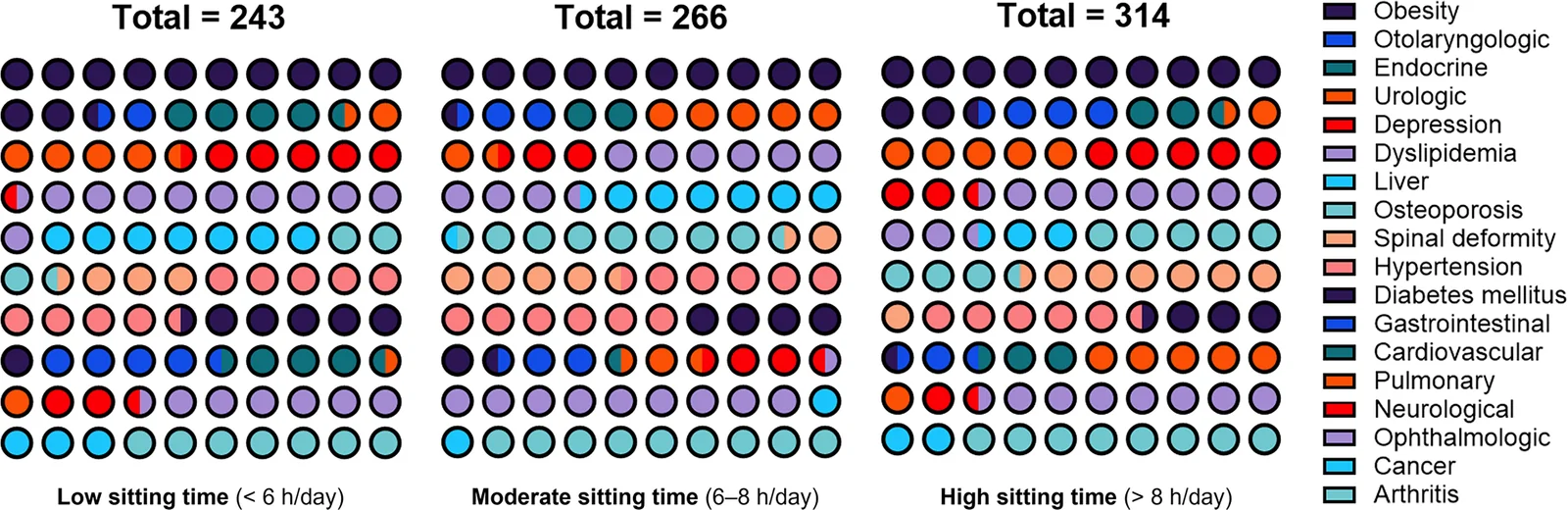
Chronic condition part of whole according to three groups. The prevalence and distribution of physician-diagnosed chronic conditions across sitting time categories (<6, 6–8, and >8 h/day) are illustrated using a 10 × 10 dot plot to visualize group-wise differences in chronic disease burden. Each colored dot represents one reported chronic condition. Colors correspond to disease categories listed in the legend. The total number above each panel represents the total number of reported chronic conditions within each sitting time group. This visualization illustrates differences in multimorbidity burden across sitting time groups and is intended for descriptive purposes rather than statistical comparison. Overall, participants with higher daily sitting time exhibited a greater burden of chronic diseases, both in terms of prevalence of individual conditions and the average number of diseases per individual.

A total of 320 older adults were screened for eligibility; after applying the inclusion and exclusion criteria, 200 participants were included in the final analysis. The older adults enrolled in this study were classified into three groups based on previously reported thresholds of sitting time: a low sitting time group (LST), a moderate sitting time group (MST), and a high sitting time group (HST), respectively. As shown in Table 1, a total of 200 participants were included in the study, with ages ranging from 65 to 96 years. The mean age was 78.7 ± 7.1 years. Age differed significantly among the three groups, increasing progressively with higher sitting time. Participants in the HST were the oldest, whereas those in the LST were the youngest. Sex distribution, monthly living expenses, and residential area showed significant group differences. In contrast, education level, spouse status, living arrangement, and self-rated health status did not differ significantly among the groups. Regarding individual chronic conditions, the prevalence of several diseases increased progressively with greater sitting time. Specifically, obesity, urologic disease, dyslipidemia, osteoporosis, spinal deformity, pulmonary disease, ophthalmologic disease, and arthritis were markedly more prevalent in the HST compared with the LST. Although formal statistical comparisons were not performed for each individual disease due to the multiple-response nature of the data, the overall pattern consistently indicated an accumulation of chronic conditions with increasing sitting time. The mean number of chronic conditions per participant increased stepwise from the LST to the MST and was highest in the HST group, indicating a large effect size. When chronic disease burden was operationalized as the mean number of diseases per participant, significant differences among groups were identified using ANCOVA (F = 7.467, *p* < 0.001, η^2^ = 0.239) (Fig. 1). Meanwhile, in-bed time, daily caloric intake, and weekly PA level were comparable across groups, with no statistically significant differences observed.

**Table 1:** Demographic and anthropometric features of participants according to sitting time category (*n* = 200).

Items	Categories	Groups			H	η^2^	
		LST (*n* = 81)	MST (*n* = 60)	HST (*n* = 59)			
**Demographics**						
Age (y)		75.6 ± 6.2^c^	79.2 ± 5.8^b^	82.4 ± 7.5^a^	30.854***	0.161
Sex	Male	44 (54.3)	18 (30.0)	19 (32.2)	10.797**	0.054
(*n*/%)	Female	37 (45.7)	42 (70.0)	40 (67.8)		
Living expenses/month	<$760	17 (21.0)	2 (3.3)	13 (22.0)	10.593**	0.042
(*n*/%)	$770–1,140	24 (29.6)	14 (23.3)	19 (32.2)		
	$1,150–1,530	20 (24.7)	22 (36.7)	11 (18.6)		
	$1,540–1,920	6 (7.4)	8 (13.3)	9 (15.3)		
	$1,920–2,300	10 (12.3)	11 (18.3)	4 (6.8)		
	≥$2,300	4 (4.9)	3 (5.0)	3 (5.1)		
Residential area	Rural	58 (71.6)	57 (95.0)	48 (81.4)	12.451**	0.063
(*n*/%)	Urban	23 (28.4)	3 (5.0)	11 (18.6)		
Education level	Illiterate	0.0	0.0	0.0	3.224	0.022
(*n*/%)	No formal schooling but literate	0.0	0.0	0.0		
	Elementary school graduate	1 (1.2)	2 (3.3)	4 (6.8)		
	Middle school graduate	2 (2.5)	0 (0.0)	4 (6.8)		
	High school graduate	28 (34.6)	16 (26.7)	17 (28.8)		
	University graduate	25 (30.9)	23 (38.3)	21 (35.6)		
	Graduate school or higher	25 (30.9)	19 (31.7)	13 (22.0)		
Marital status	With spouse	45 (55.6)	38 (63.3)	33 (55.9)	0.998	0.005
(*n*/%)	Divorced, bereaved	36 (44.4)	22 (36.7)	26 (44.1)		
Living arrangement	Living alone	40 (49.4)	29 (48.3)	26 (44.1)	0.408	0.002
(*n*/%)	Living with family	41 (50.6)	31 (51.7)	33 (55.9)		
Self-rated health status	Very good	6 (7.4)	5 (8.3)	10 (16.9)	0.531	0.004
(*n*/%)	Good	16 (19.8)	13 (21.7)	11 (18.6)		
	Fair	34 (42.0)	18 (30.0)	15 (25.4)		
	Poor	23 (28.4)	20 (33.3)	20 (33.9)		
	Very poor	2 (2.5)	4 (6.7)	3 (5.1)		
Chronic disease numbers		3.0 ± 1.1	4.4 ± 1.5	5.3 ± 1.7	66.279***	0.326
In-bed time (h/day)		6.4 ± 0.9	6.4 ± 1.0	6.1 ± 0.6	1.539	0.018
Daily meals (kcal)		1,359.4 ± 191.8	1,330.3 ± 154.3	1,300.5 ± 213.8	4.769	0.017
Activity (MET-h/week)		22.8 ± 3.2	22.2 ± 2.5	21.8 ± 3.6	5.318	0.017
Sitting time (h/day)		4.0 ± 0.9^c^	6.5 ± 0.5^b^	8.1 ± 0.2^a^	179.236***	0.873
**Body composition**						
Height (cm)		167.2 ± 6.2	165.5 ± 3.9	165.0 ± 6.4	8.227*	0.031
Body weight (kg)		71.2 ± 7.6^ab^	69.3 ± 7.2^ab^	65.5 ± 10.9^c^	7.129*	0.070
Body mass index (kg/m^2^)		25.5 ± 2.7	25.3 ± 2.7	24.0 ± 3.5	3.509	0.045
Body fat (%)		22.9 ± 4.3	23.4 ± 2.7	24.1 ± 3.0	4.417	0.019
Skeletal muscle mass		30.7 ± 5.4^a^	24.3 ± 3.6^bc^	22.6 ± 3.4^bc^	76.895***	0.404

**Notes:**

LST, low sitting time; MST, moderate sitting time; HST, high sitting time.

Self-rated health status was assessed on (1) very good, (2) good, (3) fair, (4) poor, and (5) very poor.

* *p* < 0.05.

** *p* < 0.01.

*** *p* < 0.001.

Values sharing different superscript letters (a, b, c) differ significantly between sitting-time groups according to Bonferroni-adjusted *post hoc* comparisons (*p* < 0.05).

As also shown in Table 1, significant variations in body composition were identified across the groups, with greater sitting time associated with lower height and body weight. *Post-hoc* comparisons indicated significant differences in body weight across the groups. The HST exhibited the lowest mean body weight, whereas the MST and LST showed higher mean values. However, body fat percentage and BMI did not differ significantly across the groups. Skeletal muscle mass showed a pronounced decline from the LST to the HST (*p* < 0.001), indicating a large effect size.

### Analysis of the impact of sitting time on sarcopenia

**Figure 2:**
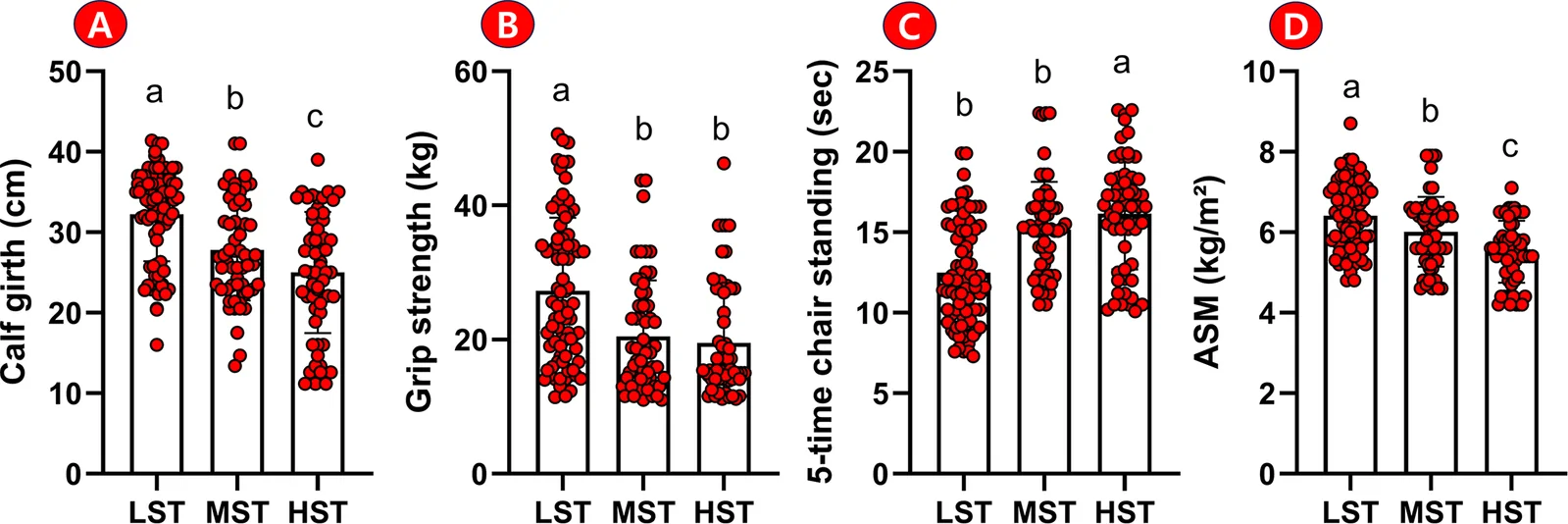
*Post-hoc* comparisons in sarcopenic parameters across sitting time groups. In *post-hoc* analyses of sarcopenic parameters, calf girth (A) showed a clear stepwise decrease with increasing sitting time. LST exhibited the largest calf circumference, followed by the MST and HST. Grip strength (B), the LST demonstrated significantly higher strength compared with both the MST and HST, suggesting that the association between sitting time and grip strength differed by sex. Five-time chair stand performance (C) worsened with increasing seat time. While the main group effect was not significant after covariate adjustment, a significant sex × group interaction was again identified, indicating sex-specific differences in functional decline associated with prolonged sitting. ASM (D), significant differences were observed across sitting time groups, with progressively lower ASM values from LST to HST. Different superscript letters (a, b, c) indicate statistically significant differences between sitting-time groups based on Bonferroni-adjusted *post hoc* comparisons following ANCOVA.

Table 2 presents the comparative analysis of sarcopenia-related indicators across sitting time groups after adjusting for age and sex. Calf girth showed a clear stepwise decrease with increasing sitting time. Participants in the LST exhibited the largest calf circumference, followed by the MST and HST (all *post hoc p* < 0.001). Group differences were highly significant with a large effect size (η^2^ = 0.362), while no sex-by-group interaction was observed, indicating a consistent pattern across sexes. For grip strength, the LST demonstrated significantly higher strength compared with both the MST and HST. Although the main effect of group was not statistically significant after adjustment, a significant sex × group interaction was observed, suggesting that the association between sitting time and grip strength differed by sex. The effect size for age remained large (η^2^ = 0.451), highlighting age as a strong determinant of muscle strength. Five-time chair stand performance worsened with increasing sitting time, with HST showing the longest completion time. While the main group effect was not significant after covariate adjustment, a significant sex × group interaction was again identified (*p* = 0.001, η^2^ = 0.069), indicating sex-specific differences in functional decline associated with prolonged sitting. Regarding ASM, significant differences were observed across sitting time groups, with progressively lower ASM values from LST to HST (*p* < 0.001). The group effect showed a small-to-moderate effect size (η^2^ = 0.235), whereas no significant sex or interaction effects were detected, suggesting that prolonged sitting is independently associated with reduced muscle mass regardless of sex. *Post-hoc* comparisons indicated significant differences in sarcopenic parameters across sitting time groups as shown in Fig. 2.

**Table 2:** Comparative analysis of sarcopenia across groups according to sitting time. ANCOVA was used to compare sarcopenic parameters across sitting time categories while controlling for age, yielding significant between-group effects. Sex was entered as a fixed factor due to its categorical nature, and Bonferroni-adjusted *post hoc* analyses were performed.

Items	Group (*n* = 200)			*p* (η²)			
	LST (*n* = 81)	MST (*n* = 60)	HST (*n* = 59)	Age	Sex	Group	Sex × Group
Calf girth (cm)	32.2 ± 5.8^a^	27.8 ± 6.3^b^	25.0 ± 7.5^c^	0.001	0.001	0.001	0.987
				(0.367)	(0.995)	(0.362)	(0.001)
Grip strength (kg)	27.3 ± 9.9^a^	20.5 ± 8.4^b^	19.5 ± 8.4^b^	0.001	0.060	0.848	0.001
				(0.451)	(0.881)	(0.149)	(0.068)
5-time standing (sec)	12.5 ± 3.2^b^	15.2 ± 3.0^b^	16.2 ± 3.5^a^	0.001	0.148	0.508	0.001
				(0.352)	(0.724)	(0.483)	(0.069)
ASM (kg/m^2^)	6.4 ± 0.9^a^	6.0 ± 0.9^b^	5.5 ± 0.8^c^	0.001	0.066	0.235	0.255
				(0.160)	(0.863)	(0.717)	(0.014)

**Notes:**

LST, low sitting time; MST, moderate sitting time; HST, high sitting time; ASM, appendicular skeletal muscle mass.

Values sharing different superscript letters (a, b, c) differ significantly between sitting-time groups according to Bonferroni-adjusted *post hoc* comparisons (*p* < 0.05).

### Analysis of the impact of sitting time on ADL

Table 3 summarizes the comparative results of ADL across sitting time groups after adjusting for age and sex. Overall, ADL performance declined progressively with increasing sitting time, with the HST consistently showing lower scores across multiple ADL domains. For basic self-care activities, significant group differences were observed in feeding, bathing, dressing, bowel control, bladder control, and toilet use. In these domains, the HST demonstrated significantly poorer performance compared with the LST, while the MST generally showed intermediate values. However, the main effect of group was not statistically significant after covariate adjustment, and the associated effect sizes were small, indicating that age accounted for a substantial proportion of variance in basic ADL function. Transfers exhibited a marked stepwise decline from LST to MST to HST, with a large age effect and a modest but significant sex × group interaction, suggesting sex-specific differences in functional decline associated with prolonged sitting. Similarly, mobility and stair climbing performance decreased with increasing sitting time, with the LST performing significantly better than the HST. No significant differences across sitting time groups were observed for grooming, indicating that highly preserved self-care activities may be less sensitive to sedentary behavior levels in community-dwelling older adults. For the ADL sum score, participants in the HST had significantly lower overall functional independence compared with those in the LST and MST. Although the main group effect did not reach statistical significance after adjustment, the large age-related effect size highlights aging as the dominant determinant of overall ADL performance, while prolonged sitting appears to exacerbate functional vulnerability.

**Table 3:** Comparative results of ADL by groups. ANCOVA was used to compare ADL across sitting time categories while controlling for age, yielding significant between-group effects. Sex was entered as a fixed factor due to its categorical nature, and Bonferroni-adjusted *post hoc* analyses were performed.

Items	Group (*n* = 200)			*p* (η^2^)			
	LST (*n* = 81)	MST (*n* = 60)	HST (*n* = 59)	Age	Sex	Group	Sex × Group
Feeding	7.8 ± 2.5^a^	7.3 ± 3.2^ab^	6.3 ± 3.2^c^	0.001	0.279	0.797	0.290
				(0.254)	(0.006)	(0.003)	(0.013)
Bathing	4.8 ± 1.1^a^	4.8 ± 1.1^a^	4.0 ± 2.0^b^	0.001	0.207	0.401	0.031
				(0.059)	(0.624)	(0.579)	(0.035)
Grooming	4.9 ± 0.8	4.9 ± 0.6	5.0 ± 0.0	0.259	0.136	0.611	0.519
				(0.007)	(0.721)	(0.311)	(0.007)
Dressing	7.6 ± 2.9^a^	7.9 ± 3.1^a^	6.4 ± 3.1^b^	0.001	0.649	0.269	0.308
				(0.102)	(0.118)	(0.674)	(0.012)
Bowels	8.5 ± 2.5^a^	8.3 ± 2.4^a^	6.9 ± 2.5^b^	0.001	0.695	0.254	0.260
				(0.098)	(0.090)	(0.696)	(0.014)
Bladder	8.5 ± 2.5^a^	8.4 ± 2.3^a^	6.9 ± 2.5^b^	0.001	0.348	0.173	0.355
				(0.105)	(0.411)	(0.769)	(0.011)
Toilet use	8.6 ± 2.3^a^	8.3 ± 2.4^a^	7.0 ± 2.5^b^	0.001	0.588	0.272	0.234
				(0.139)	(0.164)	(0.681)	(0.015)
Transfers	11.9 ± 3.1^a^	10.5 ± 2.6^b^	9.1 ± 3.7^c^	0.001	0.859	0.626	0.021
				(0.393)	(0.020)	(0.360)	(0.039)
Mobility	10.4 ± 4.4^a^	8.9 ± 3.7^ab^	7.5 ± 4.0^bc^	0.001	0.222	0.808	0.073
				(0.420)	(0.598)	(0.180)	(0.027)
Stairs	5.3 ± 3.7^a^	3.7 ± 2.9^b^	3.2 ± 3.4^b^	0.001	0.484	0.841	0.058
				(0.226)	(0.262)	(0.150)	(0.029)
ADL sum scores	78.1 ± 19.4^a^	72.9 ± 18.0^a^	62.4 ± 20.5^b^	0.001	0.838	0.386	0.163
				(0.344)	(0.026)	(0.576)	(0.019)

**Notes:**

LST, low sitting time; MST, moderate sitting time; HST, high sitting time; ADL, activities daily living.

Values sharing different superscript letters (a, b, c) differ significantly between sitting-time groups according to Bonferroni-adjusted *post hoc* comparisons (*p* < 0.05).

### Analysis of the impact of sitting time on IADL

Across nearly all IADL domains, functional independence declined markedly with increasing sitting time as shown in Table 4, with the HST consistently demonstrating the poorest performance. Significant group differences were observed for telephone use, food preparation, housekeeping, laundry, transportation, medication management, and financial management. In these domains, participants in the HST scored significantly lower than those in the LST, while the MST generally showed intermediate values. The magnitude of these differences was substantial, with large age-related effect sizes across most items, indicating that age remained the strongest determinant of IADL function. Notably, telephone use and housekeeping demonstrated significant sex × group interaction effects (*p* = 0.002 and *p* = 0.007, respectively), suggesting that the adverse impact of prolonged sitting on these cognitively and organizationally demanding tasks may differ between men and women. In contrast, shopping did not differ significantly across sitting time groups, indicating that certain community-based activities may be influenced by environmental or social support factors beyond individual sedentary behavior. For the IADL sum score, a pronounced decline was observed with increasing sitting time. Participants in the HST showed significantly lower overall IADL independence compared with both the LST and MST. Although the main group effect was modest after adjustment, the large effect size for age underscores the dominant influence of aging on higher-level functional capacity, while excessive sitting appears to amplify vulnerability to IADL dependence.

**Table 4:** Comparative results of IADL by groups. ANCOVA was used to compare IADL across sitting time categories while controlling for age, yielding significant between-group effects. Sex was entered as a fixed factor due to its categorical nature, and Bonferroni-adjusted *post hoc* analyses were performed.

Items	Group (*n* = 200)			*p* (η^2^)			
	LST (*n* = 81)	MST (*n* = 60)	HST (*n* = 59)	Age	Sex	Group	Sex × Group
Telephone	1.0 ± 0.2^a^	0.9 ± 0.3^a^	0.5 ± 0.5^b^	0.001	0.155	0.182	0.002
				(0.282)	(0.712)	(0.809)	(0.064)
Shopping	0.6 ± 0.5	0.5 ± 0.5	0.4 ± 0.5	0.001	0.171	0.676	0.243
				(0.268)	(0.675)	(0.291)	(0.015)
Food preparation	0.8 ± 0.4^a^	0.6 ± 0.5^a^	0.3 ± 0.5^b^	0.001	0.946	0.300	0.055
				(0.186)	(0.003)	(0.676)	(0.030)
Housekeeping	1.0 ± 0.2^a^	0.8 ± 0.4^a^	0.5 ± 0.5^b^	0.001	0.299	0.194	0.007
				(0.341)	(0.487)	(0.794)	(0.050)
Laundry	0.9 ± 0.3^a^	0.7 ± 0.5^b^	0.4 ± 0.5^c^	0.001	0.347	0.164	0.164
				(0.373)	(0.418)	(0.804)	(0.019)
Transportation	0.9 ± 0.3^a^	0.7 ± 0.4^a^	0.4 ± 0.5^b^	0.001	0.604	0.149	0.103
				(0.321)	(0.154)	(0.826)	(0.023)
Medications	0.8 ± 0.4^a^	0.7 ± 0.4^a^	0.4 ± 0.5^b^	0.001	0.628	0.213	0.062
				(0.150)	(0.136)	(0.764)	(0.028)
Finances	0.9 ± 0.3^a^	0.8 ± 0.4^a^	0.4 ± 0.5^b^	0.001	0.456	0.153	0.092
				(0.395)	(0.291)	(0.823)	(0.024)
IADL sum scores	6.6 ± 2.0^a^	5.8 ± 2.6^a^	3.2 ± 3.6^b^	0.001	0.328	0.186	0.017
				(0.430)	(0.448)	(0.799)	(0.041)

**Notes:**

LST, low sitting time; MST, moderate sitting time; HST, high sitting time; IADL, instrumental activities of daily living.

Values sharing different superscript letters (a, b, c) differ significantly between sitting-time groups according to Bonferroni-adjusted *post hoc* comparisons (*p* < 0.05).

### Correlations between sarcopenic parameters and functional independence

As shown in Table 5, ADL sum score showed a strong positive correlation with IADL sum score, indicating substantial overlap between basic and instrumental functional independence. ADL sum score was also moderately correlated with calf girth, grip strength, and ASM, suggesting that greater muscle mass and strength are associated with better basic daily functioning. In contrast, ADL sum score exhibited a moderate inverse correlation with 5-time chair standing, indicating that poorer lower-extremity functional performance was associated with reduced ADL independence. Similarly, IADL sum score was positively correlated with calf girth, grip strength, and ASM, while showing a negative correlation with 5-time chair standing. Notably, the magnitude of these associations was comparable to or slightly stronger than those observed for ADL sum score, underscoring the sensitivity of IADL sum score to sarcopenic impairments. Among sarcopenia-related variables, ASM was positively correlated with calf girth and grip strength and negatively correlated with 5-time chair standing.

**Table 5:** Spearman relationship among ADL, IADL, and sarcopenic variables. Spearman’s correlation (ρ) analysis was conducted to examine bivariate associations between sarcopenia components and measures of functional independence, including ADL and IADL.

		ADL sum	IADL sum	Calf girth	Grip strength	5-time stand	ASM
ADL sum	ρ	1.000	0.812**	0.453**	0.0534**	−0.478**	0.270**
IADL sum	ρ		1.000	0.403**	0.444**	−0.446**	0.299**
Calf girth	ρ			1.000	0.639**	−0.628**	0.454**
Grip strength	ρ				1.000	−0.640**	0.351**
5-time stand	ρ					1.000	−0.396**
ASM	ρ						1.000

**Notes:**

ADL, activities of daily living; IADL, instrumental activities of daily living; ASM, appendicular skeletal muscle mass.

** *p* < 0.01.

### Multiple regression analyses examining the associations of sitting time with sarcopenia-related indicators, ADL, and IADL

As shown in Table 6, sitting time was significantly associated with multiple sarcopenia-related indicators and functional independence outcomes. First, sitting time was significantly negatively associated with calf circumference, indicating that longer sitting time was related to smaller calf girth. Second, sitting time was significantly negatively associated with grip strength, suggesting reduced upper-extremity muscle strength with longer sitting time. Third, sitting time was significantly positively associated with five-time chair stand test performance time, indicating poorer lower-extremity physical performance among individuals with longer sitting time. Fourth, sitting time was significantly negatively associated with ASM, demonstrating that longer sitting time was related to lower muscle mass. Finally, sitting time was significantly negatively associated with ADL and IADL sum scores, indicating reduced independence in basic daily activities and higher-level functional activities among older adults with longer sitting time.

**Table 6:** Regression coefficients for final model. To examine the associations of sitting time with sarcopenia, ADL, and IADL, a multiple linear regression analysis was performed using a stepwise selection method. When two or more independent variables were found to be statistically significant predictors of the dependent variable, their relative contributions were compared using standardized regression coefficients (β). Multicollinearity among the independent variables was assessed by calculating variance inflation factors.

Dependent variable	B	SE	β	t	*p*	R^2^
Calf girth	−0.029	0.004	−0.442	−6.925	<0.001	0.195
Grip strength	−0.036	0.006	−0.385	−5.870	<0.001	0.148
5-time chair stand	0.015	0.002	0.465	7.396	<0.001	0.216
ASM	−0.003	0.001	−0.415	−6.427	<0.001	0.173
ADL	−0.057	0.013	−0.308	−4.549	<0.001	0.095
IADL	−0.012	0.002	−0.410	−6.324	<0.001	0.168

**Notes:**

ASM, appendicular skeletal muscle mass; ADL, activities of daily living; IADL, instrumental activities of daily living.

## Discussion

This study demonstrated that greater sitting time was associated with poorer sarcopenia-related outcomes and reduced functional independence among community-dwelling older adults, independent of age and sex. The principal findings were threefold. First, prolonged sitting time was associated with adverse demographic and health profiles, including older age, greater chronic disease, and reduced skeletal muscle mass. Second, all sarcopenic parameters were significantly impaired with increasing sitting time. Third, higher sitting time was associated with progressively lower ADL and IADL scores. Notably, PA levels did not differ significantly across the groups. These findings support the notion that sitting time represents a distinct physiological exposure, independent of moderate-to-vigorous physical activity. Together, these findings suggest that prolonged sitting time is closely associated with reduced skeletal muscle mass, impaired physical performance reflecting functional consequences of sarcopenia, and decreased functional independence, with particularly pronounced effects observed across both basic activities of daily living and higher-order functional abilities required for instrumental activities of daily living, supporting the potential role of prolonged sitting time as an important functional vulnerability factor in older adults. The results of this study are consistent with the conceptualization of sedentary behavior as a social–biological exposure rather than merely a modifiable lifestyle choice, supporting the interpretation that prolonged sitting time may reflect broader functional vulnerability in older adults (Hamilton, Ghert & Simpson, 2015).

Consistent with previous epidemiological evidence, participants with longer sitting time were older and exhibited a higher burden of chronic diseases (Biswas et al., 2015; Owen et al., 2011). Importantly, skeletal muscle mass declined markedly from the low- to high-sitting groups, with a large effect size (η^2^ = 0.404), indicating that sitting time is strongly associated with muscle quantity in older adults. In parallel, significant group differences were observed in calf girth, grip strength, and performance on the 5-time chair standing, supporting the notion that sitting time is linked to both morphological and functional dimensions of sarcopenia. Similar with previous literature, increasing sitting time was associated with progressive declines in skeletal muscle mass, strength, and physical performance (Biswas et al., 2015; Tremblay et al., 2017). Participants in the HST (>8 h/day) exhibited significantly lower ASM, reduced grip strength, smaller calf girth, and poorer chair standing performance compared with those in the LST. These findings align with experimental and observational studies demonstrating that prolonged muscle unloading and inactivity accelerate muscle protein breakdown, reduce anabolic sensitivity, and promote neuromuscular deconditioning in older adults (Breen & Phillips, 2011; Kilroe et al., 2025).

Notably, among the sarcopenia-related indicators, chair standing performance showed the strongest association with sitting time in both bivariate correlations and multivariate regression. This suggests that functional performance limitations, particularly in lower-extremity power and postural transitions, may represent a proximal determinant of sitting time. Difficulty rising from a chair may directly discourage standing and ambulation, reinforcing prolonged sitting throughout the day. From a physiological perspective, prolonged sitting posture may reduce habitual mechanical loading and neuromuscular activation of lower extremity muscles, thereby accelerating age-related declines in muscle protein synthesis and muscle quality (Dirks et al., 2016). These mechanisms may explain why individuals in the high sitting time group exhibited poorer physical performance and lower muscle mass despite no significant differences in weekly PA time across groups. This finding reinforces the concept that sitting time represents a distinct behavioral risk factor, independent of structured PA, with specific relevance to sarcopenia-related impairments (Tremblay et al., 2017).

A key contribution of this study is the demonstration that sitting time is differentially associated with basic and higher-level functional independence. While total ADL scores declined significantly with increasing sitting time, the effect size was modest compared with that observed for IADL. Moreover, ADL impairments were limited to specific items—primarily those involving mobility and physical engagement—whereas IADL impairments were widespread across nearly all domains. Specifically, IADL tasks, such as transportation use, shopping, and financial management, require greater integration of physical capacity, balance, coordination, and cognitive–motor processing than basic self-care activities assessed by ADL (Lawton & Brody, 1969). The observation that seven of eight IADL items differed significantly across groups suggests that prolonged sitting may preferentially affect functions that depend on dynamic movement and environmental interaction. This pattern is consistent with a hierarchical model of functional decline, in which higher-order activities deteriorate earlier than basic ADL as physiological reserves diminish (Mollinedo & Cancela, 2020; Stuck et al., 1999). This pattern is theoretically coherent. ADL reflects basic self-care functions that are often preserved until later stages of functional decline, whereas IADL requires greater physical capacity, cognitive integration, mobility, and social engagement (Wang et al., 2025). Prolonged sitting time may therefore preferentially impair higher-order functions that depend on sustained PA and environmental interaction. Similar gradients have been reported in longitudinal studies linking physical inactivity to earlier declines in IADL than ADL (Dunlop et al., 2015; Paterson & Warburton, 2010).

The item-level patterns observed in both ADL and IADL align closely with the impairments seen in sarcopenia-related physical performance, particularly the 5-time chair standing. Chair stand performance reflects lower-extremity strength, power, balance, and neuromuscular coordination, all of which are critical for mobility-related daily activities. The progressive worsening of chair standing performance across groups, accompanied by pronounced declines in IADL, suggests that physical performance may serve as a proximal mechanism linking sedentary behavior to functional disability. This interpretation is supported by previous longitudinal studies demonstrating that measures of lower-extremity performance are stronger predictors of disability, institutionalization, and mortality than muscle mass alone (Dent et al., 2018; Studenski et al., 2011). The present findings extend this evidence by showing that sitting time is associated with impairments in these performance-based measures, which in turn correspond to declines in functional independence.

The present study demonstrated that sitting time was significantly associated with multiple sarcopenia-related indicators and functional independence outcomes. Specifically, longer sitting time was associated with smaller calf circumference, reduced grip strength, poorer five-time chair stand performance, and lower appendicular skeletal muscle mass, suggesting consistent relationships between sedentary exposure and key components of sarcopenia. In addition, sitting time was negatively associated with both ADL and IADL scores, indicating reduced independence in both basic and higher-level daily activities among older adults with longer sitting time. Taken together, these findings suggest that sitting time may reflect a combination of musculoskeletal status and functional capacity in later life (Kumahara et al., 2025). Lower muscle mass and poorer physical performance were associated with longer sitting time at the biological level, while reduced IADL scores were associated with limitations in higher-level functional independence (Hamilton, Hamilton & Zderic, 2007). Rather than representing sedentary behavior solely as a behavioral risk factor, the present findings suggest that sitting time may be closely linked with broader age-related changes in muscle-related health and functional independence. However, because the present study employed a cross-sectional design, these associations should be interpreted as relationships rather than causal pathways.

A major strength of this study is that it comprehensively evaluated sarcopenia using the AWGS 2019 criteria while simultaneously examining both overall and item-specific components of ADL and IADL across sitting time categories, enabling a detailed assessment of how sitting time is associated with sarcopenia-related indicators and multiple domains of functional independence. Sitting lifestyle habits were assessed through self-report and the inclusion of smartphone-based activity tracking data supplemented objective information on daily movement patterns, partially mitigating recall bias. However, the cross-sectional design of this study precludes causal inference, and the findings may not be generalizable to institutionalized or severely frail older adults. In addition, although disease-specific analyses were not a primary aim of the study, statistical testing for individual diseases was limited due to the multiple-response structure of the data. These results should be interpreted in the context of the study design and its limitations, and future studies using longitudinal designs with accelerometry are needed to better clarify temporal relationships and potential causal pathways.

## Conclusions

These findings suggest a conceptual framework describing potential associations among sitting time, sarcopenia-related indicators, and functional independence. Reducing sitting time and preserving physical performance and muscle mass may therefore be critical targets for preventing functional dependence in aging populations.

## Supplemental Information

10.7717/peerj.21714/supp-1 Supplemental Information 1 Documents for evaluating the questionnaire and sitting time periods. Each data point represents raw data from 200 individuals. The groups are divided into three groups based on sedentary time: Group 1 (6 h or less), Group 2 (6–8 h), and Group 3 (8 h or more). Gender is categorized as one for male and two for female. The yellow side represents “Demographic Data,” the blue side represents “Body Composition,” the red side represents “Activities of Daily Living (ADL),” the white side represents “Instrumental Activities of Daily Living (IADL),” and the purple side represents “Sarcopenia-Related Indicators.”

10.7717/peerj.21714/supp-2 Supplemental Information 2 STROBE Documentation. The STROBE checklist of this study was used to improve the transparency, completeness, and methodological quality of reporting in observational studies, enabling readers, reviewers, and editors to critically appraise the study design, analysis, and interpretation, and to enhance reproducibility and comparability across studies.

10.7717/peerj.21714/supp-3 Supplemental Information 3 Codebook for categorical variables used in the study. Numeric codes in the raw dataset were converted into categorical factors as defined in this table.

## Additional Information and Declarations

### Data Availability

The following information was supplied regarding data availability:

The raw measurements are available in the Supplemental Files.
