# Supplementary material for: Reducing sitting time is essential for preserving sarcopenia and functional independence with aging: a comparative cross-sectional study
Source: PeerJ. 2026 Sep 8;14:e21714. doi: 10.7717/peerj.21714 (PMC13564411; doi:10.7717/peerj.21714)
Supplement: Supplementary file 1 [file peerj-14-21714-s002.pdf]

STROBE Statement—checklist of items that should be included in reports of observational studies

	Item No.	Recommendation	Page No.	Relevant text from manuscript
**Title and abstract**	1	(*a*) Indicate the study's design with a commonly used term in the title or the abstract	1	Reducing sitting time is essential for preserving sarcopenia and functional independence with aging: A cross-sectional cohort study
		(*b*) Provide in the abstract an informative and balanced summary of what was done and what was found	2	Longer sitting duration was associated with poorer functional independence and sarcopenic functional impairment in older adults, even after adjustment for age and sex.
**Introduction**				
Background/rationale	2	Explain the scientific background and rationale for the investigation being reported	3	…although prolonged sitting has emerged as a major risk factor contributing to functional decline in older adults, the extent to which sitting time affects sarcopenic indices and functional independence, as well as the strength and nature of the interrelationships among these variables, remains inconclusive.
Objectives	3	State specific objectives, including any prespecified hypotheses	3	…this study investigated the effects of daily sitting time on sarcopenia and functional independence in older adults and analyzed the associations among these variables.
**Methods**				
Study design	4	Present key elements of study design early in the paper	4	This study employed a cross-sectional cohort design…
Setting	5	Describe the setting, locations, and relevant dates, including periods of recruitment, exposure, follow-up, and data collection	4	From November 1, 2024, to February 28, 2025, older adults aged 65 years and older residing

				in a community facility in Seoul, Korea (Seoul Seniors Tower) were recruited. After obtaining written informed consent, participants completed a demographic questionnaire, and assessments were conducted for body composition, ADL and IADL, as well as sarcopenia-related variables.
Participants	6	(*a*) *Cohort study*—Give the eligibility criteria, and the sources and methods of selection of participants. Describe methods of follow-up<br>*Case-control study*—Give the eligibility criteria, and the sources and methods of case ascertainment and control selection. Give the rationale for the choice of cases and controls<br>*Cross-sectional study*—Give the eligibility criteria, and the sources and methods of selection of participants	4	Eligible participants were individuals who could walk independently with or without assistive devices, had not participated in supervised exercise or rehabilitation programs for more than six months, and were able to complete all required physical tests and self-report questionnaires.
		(*b*) *Cohort study*—For matched studies, give matching criteria and number of exposed and unexposed<br>*Case-control study*—For matched studies, give matching criteria and the number of controls per case	4	This study employed a cross-sectional cohort design without individual matching; therefore, no matching criteria were applied, and participants were classified according to exposure status.
Variables	7	Clearly define all outcomes, exposures, predictors, potential confounders, and effect modifiers. Give diagnostic criteria, if applicable	4	After obtaining written informed consent, participants completed a demographic questionnaire, and assessments were conducted for body composition, ADL and IADL, as well as sarcopenia-related variables.
Data sources/ measurement	8*	For each variable of interest, give sources of data and details of methods of assessment (measurement). Describe comparability of assessment methods if there is more than one group	4	Demographic characteristics related to participants' daily living were collected using standardized questionnaires. In addition, ADL and IADL were

				assessed using structured questionnaires. In contrast, body composition and sarcopenia-related variables were measured according to established assessment protocols. The specific measurement procedures are described below.
Bias	9	Describe any efforts to address potential sources of bias	4-5	To minimize potential sources of bias, standardized protocols were applied for all measurements. Trained assessors conducted physical assessments using the same procedures for all participants. Self-reported questionnaires were administered in a structured manner to reduce information bias.
Study size	10	Explain how the study size was arrived at	4	Assuming a small-to-moderate effect size ($f^2 = 0.10$), an α level of 0.05, and a statistical power of 0.80, the minimum required sample size was estimated to be 114 participants. To account for potential missing data and to ensure sufficient power for nonparametric group comparisons and item-level analyses, a larger sample size was targeted.

Continued on next page

Quantitative variables	11	Explain how quantitative variables were handled in the analyses. If applicable, describe which groupings were chosen and why	7	Continuous variables are presented as means ± standard deviations, and categorical variables are presented as frequencies and percentages [n (%)] and were compared using the χ^2 test or Fisher's exact test, as appropriate.
Statistical methods	12	(*a*) Describe all statistical methods, including those used to control for confounding	7	All statistical analyses were performed using IBM SPSS Statistics version 27.0 (IBM Corp., Armonk, NY, USA) and GraphPad Prism 10.6.1 (La Jolla, CA, USA).
		(*b*) Describe any methods used to examine subgroups and interactions	7	The normality of continuous variables was assessed using the Kolmogorov–Smirnov test. Prior to the main analyses, differences in baseline characteristics among the three groups were examined using the Kruskal–Wallis test. An analysis of covariance (ANCOVA) was used to compare body composition and sarcopenic parameters across sitting time categories while controlling for age, yielding significant between-group effects. Sex was entered as a fixed factor due to its categorical nature, and Bonferroni-adjusted post hoc analyses were performed.
		(*c*) Explain how missing data were addressed	—	Not applicable
		(*d*) *Cohort study*—If applicable, explain how loss to follow-up was addressed<br>*Case-control study*—If applicable, explain how matching of cases and controls was addressed<br>*Cross-sectional study*—If applicable, describe analytical methods taking account of sampling strategy	8	Given the cross-sectional design, analytical methods accounted for potential confounding by adjusting for age and sex, while no additional sampling-weighted analyses were required.
		(*e*) Describe any sensitivity analyses	8	No additional sensitivity analyses were conducted beyond the primary analyses adjusted for age and sex.
**Results**				

Participants	13*	(a) Report numbers of individuals at each stage of study—eg numbers potentially eligible, examined for eligibility, confirmed eligible, included in the study, completing follow-up, and analysed	8	A total of 200 participants were included in the study, with ages ranging from 65 to 96 years. The mean age was 78.7 ± 7.1 years. The sample comprised 81 men and 119 women. Specifically, the LST included 81 (44 men and 37 women), the MST comprised 60 (18 men and 42 women), and the HST consisted of 59 (19 men and 40 women).
		(b) Give reasons for non-participation at each stage	—	Not applicable
		(c) Consider use of a flow diagram	—	The participant selection process could have been presented using a CONSORT flow chart, but it was not presented because it was not an RCT study.
Descriptive data	14*	(a) Give characteristics of study participants (eg demographic, clinical, social) and information on exposures and potential confounders	8	Age differed significantly among the three groups, increasing progressively with higher sitting time. Participants in the HST were the oldest, whereas those in the LST were the youngest. Sex distribution, monthly living expenses, and residential area showed significant group differences.
		(b) Indicate number of participants with missing data for each variable of interest	—	Not applicable
		(c) *Cohort study*—Summarise follow-up time (eg, average and total amount)	—	Not applicable
Outcome data	15*	*Cohort study*—Report numbers of outcome events or summary measures over time	8	Outcome data were summarized using means and standard deviations for continuous variables (e.g., body composition, sarcopenia-related indicators, ADL and IADL scores) and frequencies with percentages for categorical variables.
		Case-control study—Report numbers in each exposure category, or summary measures of exposure	—	Not applicable
		Cross-sectional study—Report numbers of outcome events or summary measures	8	Outcome data were summarized using means and standard

				deviations for continuous variables (e.g., body composition, sarcopenia-related indicators, ADL and IADL scores) and frequencies with percentages for categorical variables.
Main results	16	(*a*) Give unadjusted estimates and, if applicable, confounder-adjusted estimates and their precision (eg, 95% confidence interval). Make clear which confounders were adjusted for and why they were included	8	…age and sex were included as confounders a priori due to their well-established associations with muscle-related outcomes and functional independence.”
		(*b*) Report category boundaries when continuous variables were categorized	9	Daily sitting time was categorized into low, moderate, and high groups based on predefined cut-off values, while sarcopenia status was categorized according to established diagnostic criteria with specific cut-off values applied for each category.
		(*c*) If relevant, consider translating estimates of relative risk into absolute risk for a meaningful time period	—	Not applicable

Continued on next page

Other analyses	17	Report other analyses done—eg analyses of subgroups and interactions, and sensitivity analyses	10-12	Overall, ADL performance declined progressively with increasing sitting time, with the HST consistently showing lower scores across multiple ADL domains.<br>Across nearly all IADL domains, functional independence declined markedly with increasing sitting time, with the HST consistently demonstrating the poorest performance.<br>Using a stepwise selection procedure, only the 5-time chair standing, ASM, and IADL sum score were retained in the final significant model.
**Discussion**				
Key results	18	Summarise key results with reference to study objectives	12	This study demonstrated that greater sitting time was associated with poorer sarcopenia-related outcomes and reduced functional independence among community-dwelling older adults, independent of age and sex.
Limitations	19	Discuss limitations of the study, taking into account sources of potential bias or imprecision. Discuss both direction and magnitude of any potential bias	15	However, the cross-sectional design of this study precludes causal inference, and the findings may not be generalizable to institutionalized or severely frail older adults. In addition, although disease-specific analyses were not a primary aim of the study, statistical testing for individual diseases was limited due to the multiple-response structure of the data. Furthermore, because cognitive function was not directly assessed, residual confounding by cognitive status cannot be excluded, particularly for IADL outcomes that require executive function and decision-making.

Interpretation	20	Give a cautious overall interpretation of results considering objectives, limitations, multiplicity of analyses, results from similar studies, and other relevant evidence	15	The findings suggest that prolonged sitting time may be an important behavioral factor associated with sarcopenia-related decline and loss of functional independence in older adults. These results should be interpreted in the context of the study design and its limitations.
Generalisability	21	Discuss the generalisability (external validity) of the study results	15	However, the cross-sectional design of this study precludes causal inference, and the findings may not be generalizable to institutionalized or severely frail older adults.
**Other information**				
Funding	22	Give the source of funding and the role of the funders for the present study and, if applicable, for the original study on which the present article is based	—	Not applicable

*Give information separately for cases and controls in case-control studies and, if applicable, for exposed and unexposed groups in cohort and cross-sectional studies.

Note: An Explanation and Elaboration article discusses each checklist item and gives methodological background and published examples of transparent reporting. The STROBE checklist is best used in conjunction with this article (freely available on the Web sites of PLoS Medicine at http://www.plosmedicine.org/, Annals of Internal Medicine at http://www.annals.org/, and Epidemiology at http://www.epidem.com/). Information on the STROBE Initiative is available at www.strobe-statement.org.
